# Spatiotemporal Patterns in pCO_2_ and Nutrient Concentration: Implications for the CO_2_ Variations in a Eutrophic Lake

**DOI:** 10.3390/ijerph191912150

**Published:** 2022-09-25

**Authors:** Jie Xu, Zheng Zhou, Jie Chen, Haihua Zhuo, Jie Ma, Yunbing Liu

**Affiliations:** 1 Yangtze River Basin Ecological Environment Monitoring and Scientific Research Center, Yangtze River Basin Ecological Environment Supervision and Administration Bureau, Ministry of Ecology and Environment, Wuhan 430010, China; 2 Nanjing Institute of Environment Sciences, Ministry of Ecology and Environment, Nanjing 210023, China

**Keywords:** Taihu Lake, nutrient concentrations, CO_2_ dynamics, eutrophication, external inputs

## Abstract

Lakes are considered sentinels of terrestrial environmental change. Nevertheless, our understanding of the impact of catchment anthropogenic activities on nutrients and the partial pressure of carbon dioxide (pCO_2_, an important parameter in evaluating CO_2_ levels in water) is still restrained by the scarcity of long-term observations. In this study, spatiotemporal variations in nutrient concentrations (total nitrogen: TN, total phosphorus: TP, nitrate: NO_3_^−^–N, and ammonium: NH_4_^+^–N) pCO_2_ in Taihu Lake were analyzed from 1992 to 2006, along with the gross domestic product (GDP) and wastewater discharge (WD) of its catchment. The study area was divided into three zones to characterize spatial heterogeneity in water quality: the inflow river mouth zone (Liangxi River and Zhihugang River), transition zone (Meiliang Bay), and central Taihu Lake, respectively. It is abundantly obvious that external nutrient inputs from the catchment have a notable impact on the water parameters in Taihu Lake, because nutrient concentrations and pCO_2_ were substantially higher in the inflow river mouth zone than in the open water of Meiliang Bay and central Taihu Lake. The GDP and WD of Taihu Lake’s catchment were significantly and positively correlated with the temporal variation in nutrient concentrations and pCO_2_, indicating that catchment development activities had an impact on Taihu Lake’s water quality. In addition, pCO_2_ was negatively correlated with chlorophyll a and the saturation of dissolved oxygen, but positively correlated with nutrient concentrations (e.g., TN, TP, and NH_4_^+^–N) in inflow river mouth zone of Taihu Lake. The findings of this study reveal that the anthropogenic activities of the catchment not only affect the water quality of Taihu Lake but also the CO_2_ concentrations. Consequently, catchment effects require consideration when modeling and estimating CO_2_ emissions from the extensively human-impacted eutrophic lakes.

## 1. Introduction

Lakes have been a hot-spot for environmental studies, notably investigations of anthropogenic eutrophication, nutrient cycling (e.g., nitrogen, phosphorus, and carbon), and carbon dioxide emissions (CO_2_) [1,2,3,4,5,6]. Inland lakes are sensitive to external changes induced by anthropogenic activities in their surrounding catchments, and are often characterized as sentinels of terrestrial environmental changes [7,8,9]. Nonetheless, the understanding of the impact of the catchment’s anthropogenic activities on nutrient and CO_2_ concentrations keeps limited by a lack of long-term observations.

Eutrophication is a natural process in the development of lake ecosystem [10]. However, the intensification of anthropogenic activities (e.g., land use change, agricultural fertilization, industrial development, and population growth) has tremendously exacerbated lake eutrophication, which has resulted in the degradation of water quality and harmful algal blooms in lakes [11,12,13]. However, reductions in nutrients (e.g., in sewage discharge and agricultural fertilizer) at the catchment scale significantly enhance the quality of surface water. Previous studies have revealed that environmental investment can enhance inland water quality in China [6,14]. Tong et al., (2020, 2017) realized that advancements in municipal wastewater treatment reduced phosphorus concentrations and altered the nitrogen to phosphorus ratio in Chinese lakes [15,16]. These studies have demonstrated a friendly association between inland water quality and anthropogenic activities in catchments. Regrettably, little consideration has been given to the catchment’s anthropogenic influence on CO_2_ variations in inland water.

Highly human-impacted eutrophic aquatic ecosystems vary in their nutrient concentrations, dissolved organic matter quality, primary productivity, and degree of watershed development [17,18,19,20], which can potentially exert an influence on carbon cycling in lakes (e.g., carbon transformation, burial, and CO_2_ emissions) [21,22]. Recently, the impact of eutrophication on lake CO_2_ variability has received increasing awareness and interest [23,24,25]. On the one hand, high concentrations of available nutrients induce high primary production in eutrophic lakes, which results in the absorption of organic carbon and results in the undersaturation of CO_2_ in lakes [17,26,27,28]. On the other hand, high nutrient levels can increase CO_2_ production through stimulating respiration [29], and the organic matter derived from algae can be decomposed rapidly and make a significant contribution to CO_2_ production in eutrophic lakes [23,30]. The closely-linked processes lead to increasing complexity in understanding the eutrophication effects on CO_2_ variability in lakes. Therefore, for a greater understanding of the connection between lake eutrophication and CO_2_ variability, additional research is required.

Over the past several decades, China has experienced rapid economic development, but this economic growth has come at the expense of the environment quality [31,32]. Land use intensification and rapid urbanization have resulted in increasing wastewater discharge (WD) from watersheds to inland waters [33,34]. Due to intensifying pace of urbanization and the expansion of agricultural production, Taihu Lake, the third largest freshwater lake in China, has shifted from a mesotrophic state to a hypereutrophic state since the 1960s [12], and the frequency of cyanobacterial blooms has expanded since the 1980s [35]. To prevent the continuous deterioration of the water quality of Taihu Lake, the Chinese government has employed strict measures to control external nutrient inputs from the surrounding catchment since 1996 [12]. Therefore, changes in the water quality of Taihu Lake have been affected in part by the economic and social development of its catchment. Taihu Lake has been a typical research site for multidisciplinary scientific researchers considering eutrophication and its eco-environmental effects [12]. Notwithstanding, little focus has been placed on the drivers of CO_2_ variability, restricting our ability to comprehend the relationship between eutrophication and CO_2_ variation in Taihu Lake. Although a variety of studies have investigated the spatial and temporal dynamics of nutrient concentrations in Taihu Lake [14,24,25,36], the relationships between nutrient concentration, CO_2_ dynamics, and watershed development are still incompletely understood.

In this study, quindecinnial observations of water quality in Taihu Lake were made, and indicators of development in its catchment were collected, including gross domestic product (GDP) and WD [6]. Spatiotemporal patterns of CO_2_ and primary water parameters in Taihu Lake were analyzed. Meanwhile, their relationships with GDP and WD were characterized. The principal objectives of this study were to (1) clarify the underlying mechanism of spatiotemporal variations of CO_2_ and nutrient concentrations and (2) elucidate the importance of catchment’s anthropogenic activities (GDP and WD) on regulating CO_2_ and nutrient concentrations in Taihu Lake from 1992 to 2006. Obviously, the purpose of this research was to produce data that will facilitate the future management of both water quality and CO_2_ emissions in eutrophic lakes, as well as to fill the gap between the development of the watershed and the dynamics of the nutrient and CO_2_ concentrations in Taihu Lake.

## 2. Materials and Methods

### 2.1. Taihu Lake

Taihu Lake is located in the southern portion of the Yangtze River delta (subtropical zone, 30°55′40″–31°32′58″ N; 119°52′32″–120°36′10″ E), which has an average depth and surface area of 1.89 m, and 2338.1 km^2^, respectively [12]. Taihu Lake experiences a strong seasonality, shown as a cold and dry winter and a hot and humid summer. The annual average air temperature and precipitation are 14.9–16.2 °C and 1177 mm yr^−1^ in the catchment of Taihu Lake, respectively. The annual average evapotranspiration is around 822 mm in the surface water of Taihu Lake. Taihu Basin is characterized by dense river networks and contains 172 rivers and channels. In addition, its catchment is surrounded by remarkably industrialized and densely populated areas (1654 per km^−2^ in 2018) and cities in China, such as Wuxi, Suzhou, Changzhou, and Shanghai.

**Figure 1:**
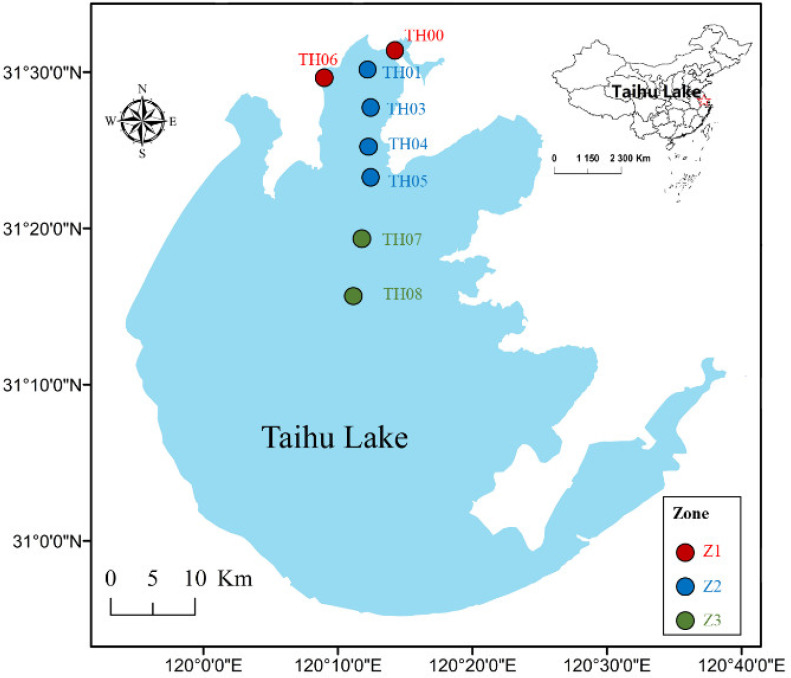
Geolocations of sample sites in Taihu Lake. TH00 and TH06 located in the inflow river mouth zone (z1, Liangxi River and Zhihugang River), TH01–TH05 located in Meiliang Bay (z2), and TH07–TH08 located in the central lake (z3).

In this study, quindecinnial observations of water parameters at eight sites from 1992 to 2006 were collected (Figure 1). To characterize spatial patterns in water quality, the research area was split up into three distinct zones: the inflow river zone (z1, Liangxi River and Zhihugang River, sites TH00 and TH06), the transition zone in Meiliang Bay (z2, sites TH01, TH03, TH04, and TH05), and the central lake (z3, sites TH07 and TH08).

### 2.2. Data Collection and Preliminary Analysis

Water physicochemical and biological parameters were acquired from the Lake–Watershed Science Data Centre, National Earth System Science Data Sharing Infrastructure, National Science & Technology Infrastructure of China (http://lake.geodata.cn (accessed on 29 January 2020)). Taihu Laboratory for Lake Ecosystems Research has been keeping track of a number of Taihu Lake’s parameters since 1992, including its physiochemical, biological, hydrological, and climatic conditions [37]. Monthly water quality data from 1992 to 2006 were analyzed from the depth-integrated water samples, including the pH, total nitrogen (TN), total phosphorus (TP), chlorophyll a (Chl–a), orthophosphate (PO_4_^3−^–P), dissolved oxygen (DO), ammonium (NH_4_^+^–N), nitrate (NO_3_^−^–N), total alkalinity (TA), water temperature (T), and electric conductivity (EC). pH was measured with a calibrated electrode using standard buffer solution. DO, T, and EC were measured by a multi-parameter probe. Chl–a concentrations were determined spectrophotometrically after extraction using ethanol [38]. TN and TP were analyzed spectrophotometrically after a combined persulfate digestion method. NH_4_^+^–N, NO_3_^−^–N, and PO_4_^3−^–P were measured with a spectrophotometer. Additionally, annual GDP and WD data for Wuxi city between 1992 and 2006 were collected from the Statistical Yearbook and Annual Report on the Environment of Wuxi, respectively.

### 2.3. Calculations of pCO_2_ and DO Saturation

In this study, monthly pCO_2_ was computed using CO_2_SYS software based on water temperature, pH, and TA [39]. pCO_2_ was calculated using the water temperature, pH, and TA as described in several studies [2,14,24,40], specific details regarding the calculation method are provided in the Supplementary Materials (SM, Text S1). Several researchers have noted that the calculations of pCO_2_ can be overestimated when the pH is lower than 7.0 [40]. However, in our dataset, the pH of 98% of the samples was higher than 7.5 (the pH was below 7 for only three samples), which indicates that the pCO_2_ calculations were robust. Likewise, the saturations of CO_2_ and DO (S_DO_) were calculated, and the detailed methods are outlined in the SM (Text S2).

### 2.4. Statistical Analysis

A Shapiro–Wilk test was utilized to assess whether the data fit a normal distribution prior to conducting statistical analyses. The non-parametric Kruskal–Wallis test was used to scrutinize variations in important water parameters (pH, TA, S_DO_, EC, TN, NH_4_^+^–N, TP, and Chl–a) in the three zones. The local polynomial regression (LOESS) procedure was used to visualize monthly dynamics of pCO_2_ and the Chl–a concentration based on monthly means, and seasonal differences in pCO_2_ and Chl–a in three zones were identified using the Kruskal–Wallis test method. To explore the factors affecting pCO_2_ in Taihu Lake, principal component analysis (PCA) was performed using standardized water parameters that may correlated to CO_2_ concentrations, including TA, EC, TN, TP, NH_4_^+^–N, and PO_4_^3−^–P. The correlations between pCO_2_ and water parameters were calculated utilizing Spearman’s rank correlation coefficient, and the level of statistical significance was set at *p* 0.05. R, version 3.5.1, was used to conduct all statistical analyses [41].

## 3. Results

### 3.1. Spatiotemporal Variations in Water Parameters

**Figure 2:**
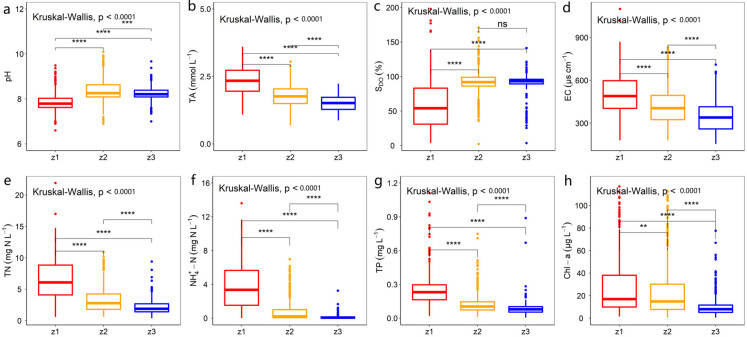
Spatial patterns in the key water parameters in different zones of Taihu Lake (non-parametric Kruskal–Wallis method). (**a**) pH; (**b**) TA: total alkalinity; (**c**) S_DO_: DO saturation; (**d**) EC: electric conductivity; (**e**) TN: total nitrogen; (**f**) NH_4_^+^–N: ammonium; (**g**) TP: total phosphorus; (**h**) Chl–a: chlorophyll a. **, ***, and **** respectively indicate the difference is significant at the *p* < 0.01, *p* < 0.001, and *p* < 0.0001 level, while ns indicated that the difference was non-significant. The numbers of above parameters were presented in Table S2 (see SM). The top and bottom of the boxes indicate the 75% and 25% confidence intervals, respectively; the horizontal line within the box is the median. The values of the 10th and 90th percentiles are represented by the upper and lower whiskers, respectively, while the dots indicate the value outside this range.

From 1992 to 2006, there was considerable spatial heterogeneity in the main water parameters of Taihu Lake (Figure 2). Results revealed that pH and S_DO_ were drastically lower in z1 (river mouth) than in z2 and z3 (*p* < 0.01, Figure 2a,c), and values of TA and EC were considerably higher in z1 (*p* < 0.01, Figure 2b,d). Specifically, it was observed that S_DO_ in z1 were significantly lower than z2 and z3. The nutrient concentrations in the three zones of Taihu Lake were also spatially heterogeneous. Specifically, the TN, NH_4_^+^–N, and TP concentrations were substantially higher in z1 than in z2 and z3 (*p* < 0.01) compared with z3 (Figure 2e–g). The concentrations of Chl–a were markedly higher in z1 and z2 than in z3 (Figure 2h).

**Figure 3:**
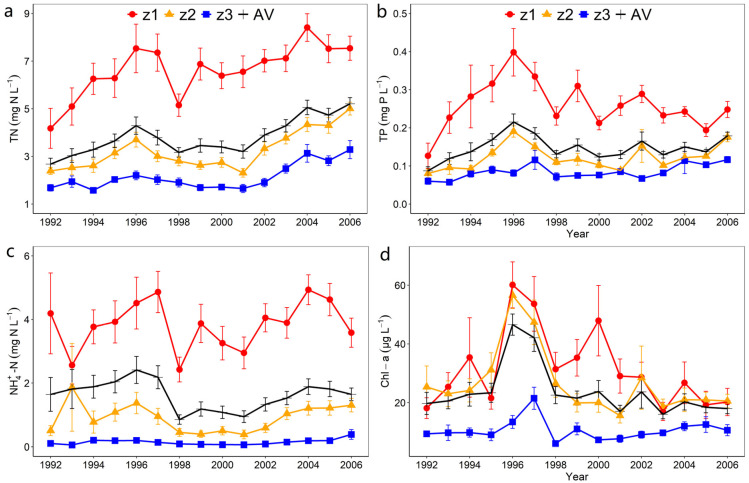
Annual concentrations (mean ± SE) of (**a**) TN, (**b**) TP, (**c**) NH_4_^+^–N, and (**d**) Chl–a concentrations from 1992 to 2006 in different zones. AV denotes the total average value of the eight sites.

A finding was formed that annual dynamics of nutrient and Chl-a concentrations were characterized by high spatiotemporal heterogeneities in Taihu Lake during 1992–2006 period (Figure 3). The annual mean concentrations of TN, NH_4_^+^–N, TP, and Chl–a were higher in z1 than in z2 and z3, and were the lowest in z3. The average concentrations of TN, NH_4_^+^–N, and TP in z1 during 1992–2006 were 6.6 ± 0.7 mg N L^−1^ (mean ± SE), 3.8 ± 0.6 mg N L^−1^, and 0.3 ± 0.03 mg L^−1^, respectively, which were nearly twice as high as those in the overall average (AV) (Figure 3a–c). The mean Chl–a concentration in z1 during 1992–2006 was 31.2 ± 6.5 μg L^−1^, which outperformed AV by 1.2 times. From the annual perspective, the concentrations of TN, NH_4_^+^–N, and TP displayed similar trends: a gradual increase from 1992 to 1996, and followed by a decline until 2000. The peak value of Chl–a concentration was found in 1996 (z1, z2) and 1997 (z3), and the Chl–a concentrations decreased from 1996 to 1998, and then fluctuated during 1998–2006 (Figure 3d). Furthermore, a co–variation in the TN and Chl–a concentrations was also observed in all three zones of Taihu Lake prior to 2000.

### 3.2. Monthly and Seasonal Patterns in pCO_2_ and Chl–a

**Figure 4:**
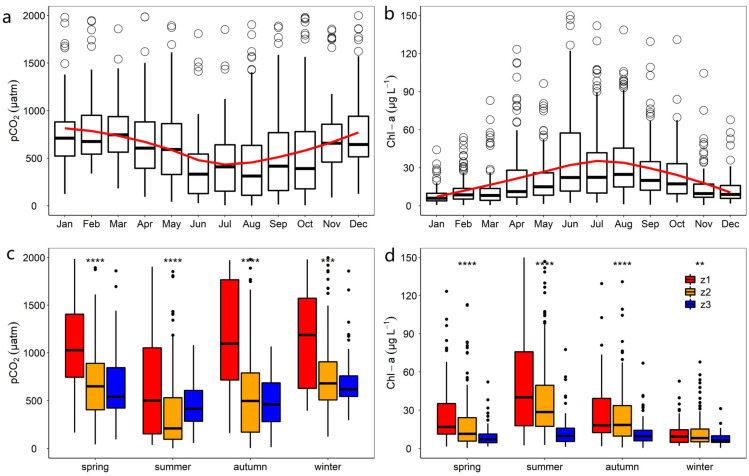
Monthly and seasonal patterns in pCO_2_ (**a**,**c**) and Chl–a (**b**,**d**) concentration from 1992 to 2006. Smooth lines in red represent the trend based on the local polynomial regression (LOESS) procedure. The top and bottom of the boxes indicate the 75% and 25% confidence intervals, respectively; the horizontal line within the box is the median. **, ***, and **** indicate the difference is significant at the *p* < 0.01, *p* < 0.001 and *p* < 0.0001 level, respectively. The values of the 10th and 90th percentiles are represented by the upper and lower whiskers, respectively, while the dots indicate the value outside this range. Kruskal–Wallis test method was utilized to identify the seasonal differences of pCO_2_ and Chl–a in different zones of Taihu Lake.

During the past 15 years, the monthly variations of pCO and Chl–a concentrations generally followed an inverse trend (Figure 4). pCO_2_ exhibited high values at the beginning and end of the year, but were low in the middle of the year (Figure 4a). pCO_2_ was highest and lowest in January and August, with mean values of 1928.1 ± 227.1 μatm, and 807.7 ± 94.6 μatm, respectively. The highest and lowest Chl–a concentrations were observed in August and January, with mean values of 36.4 ± 2.9 μg L^−1^ and 8.1 ± 0.7 μg L^−1^, respectively (Figure 4b).

Clear seasonal patterns in pCO_2_ and Chl–a in different zones of Taihu Lake were observed (Figure 4c,d). In general, pCO_2_ was higher during the winter and spring, and lower during the summer and autumn. Chl–a concentrations were highest during the summer and lowest during the winter. z1 had considerably higher pCO_2_ and Chla concentrations than the other zones (*p* < 0.01).

### 3.3. Long-Term Dynamics of pCO_2_ in Taihu Lake

**Figure 5:**
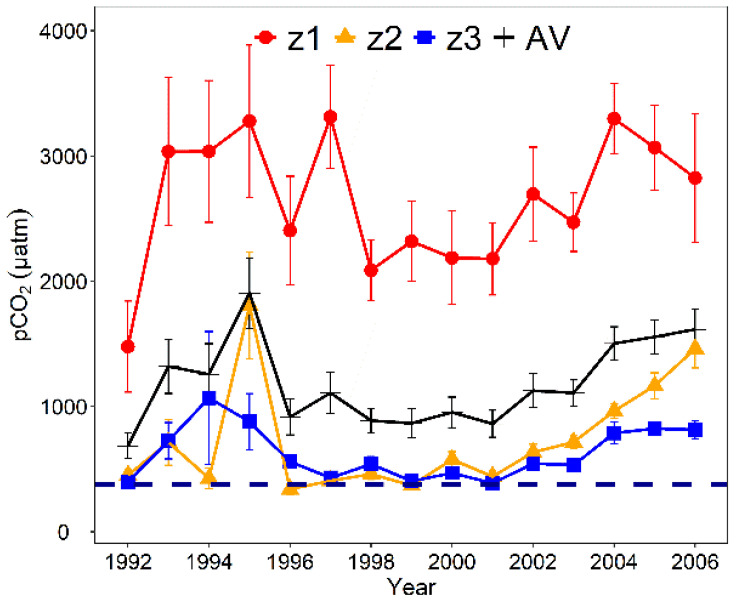
Dynamics of pCO_2_ (mean ± SE) in Taihu Lake from 1992 to 2006 in three zones. AV represent the average values of three zones, the dashed blue line indicates the partial pressure of CO_2_ in the atmosphere (380 μatm, see Figure S1).

The annual dynamics of pCO_2_ in Taihu Lake indicated that CO_2_ achieved supersaturation from 1992 to 2006, causing CO_2_ to be released from the water into the atmosphere (Figure 5). Compared with Meiliang Bay (z2) and central Taihu Lake (z3), the inflow river mouth zone (z1) exhibited higher potential for CO_2_ emissions. The total average pCO_2_ peaked in 1995, and gradually increased from 1998 to 2006.

### 3.4. Principal Component Analysis

**Figure 6:**
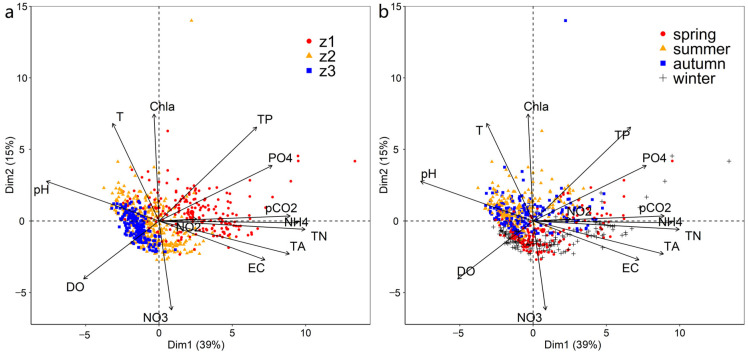
Principal component analysis (PCA) of pCO_2_ and key water variables: (**a**) spatial patterns; (**b**) seasonal patterns.

PCA was performed on the water parameters and pCO_2_ in Taihu Lake, while spatial and temporal (seasonal) information was included (Figure 6, Table S3). Two principal components accounted for 54% of the variance (Dim1: 39%, Dim2: 15%). pCO_2_, EC, TA, and nutrients including TN, TP, NH_4_^+^–N, and PO_4_^3−^–P loaded positively. However, pH and DO load negatively on the first principal component (Dim1). Water temperature and Chl–a loaded positively, and DO, NO_3_^−^–N, TP, and PO_4_^3−^–P loaded negatively on the second principal component (Dim2). Dim1 captured spatial heterogeneity in the water parameters among different zones of Taihu Lake, whereas Dim2 captured seasonal variation (namely, the contrast between the spring–winter and summer–autumn periods).

### 3.5. Correlations between pCO_2_ and Water Parameters

A non–parametric Spearman’s rank correlation analysis revealed that pCO_2_ was significantly correlated with multiple water parameters (Table 1). pCO_2_ in Taihu Lake were significantly and positively correlated to nutrient concentrations (including TN, TP, NH_4_^+^–N, and PO_4_^3−^–P) and EC (*p* < 0.01), but negatively correlated to Chl–a, DO, and S_DO_ (*p* < 0.01). Even though correlations between pCO_2_ and water parameters fluctuated among the three zones (e.g., pCO_2_ vs. NO_3_^−^–N, PO_4_^3−^–P, and Chl–a), significant positive correlations of pCO_2_ with TN, NH_4_^+^–N, and EC were observed in various zones.

## 4. Discussion

### 4.1. Spatiotemporal Variation in Key Water Parameters in Taihu Lake

Spatial variation in the key water parameters in the three zones proves that external nutrient loadings are affected by activities in the catchment of Taihu Lake, as nutrient concentrations (e.g., TN, TP, and NH_4_^+^–N) were significantly higher in the inflow river mouth zone compared with the other two zones (Figure 2e–g). The spatial patterns of water parameters in Taihu Lake emphasized the importance of external inputs from the inflow river. This outcome is even further supported by the EC values in the various zones (Figure 2d), as EC is convinced to be a signal of the effect of external pollution on nutrient concentrations in Taihu Lake.

Temporal dynamics in the annual nutrient concentrations in Taihu Lake indicated the extent to which anthropogenic activities in the catchment affect the nutrient concentration in Taihu Lake. The external loadings of TN and TP in Taihu Basin have increased approximately 2.8 and 2 times from 1960 to 1988 (from 10,000 t TN yr^−1^ and 1000 t TP yr^−1^ to 28,000 t TN yr^−1^ and 2000 t TP yr^−1^) [36], respectively. Accordingly, increases in the concentrations of TN and TP in Taihu Lake were observed from 1992 to 1996 (Figure 3a,b). After 1990, Qin et al. (2007) recommended that rapid industrialization and urbanization in the Taihu Basin have led to a decline in the water quality of Taihu Lake [12]. A Baxe Lake in Spain [42], Chaohu Lake in China [43], Indian River Lagoon in the United States [44], and the Black Burn and Lead Burn catchments in Scotland [45] are illustrations of other watersheds where anthropogenic pressure has been demonstrated to affect the health of inland aquatic ecosystems. Significant positive correlations were observed between indicators of basin development (GDP and WD) and average yearly concentrations of nutrients (Figure S3a,b,d,e), reflecting that human activities influence water quality fluctuations. Accordingly, both observed spatial and temporal variations in nutrient concentrations indicated that external inputs from the catchment had a substantial impact on nutrient concentrations in Taihu Lake between 1992 and 2006.

### 4.2. Spatiotemporal Patterns of pCO_2_ in Taihu Lake

High CO_2_ concentrations were observed in the river mouth zone (Figure 2 and Figure 5). As Taihu Lake is surrounded by industrialized and densely populated cities, human–driven external pollutant inputs (e.g., nutrients and organic and inorganic carbon) are critical drivers of spatial patterns of pCO_2_ [4,24,46,47]. Earlier studies have indicated that external dissolved inorganic carbon inputs can cause lakes to act as CO_2_ sources [21,46,48]. Moreover, external organic carbon inputs affect pCO_2_ in lakes by increasing respiration rates [4,49]. The significant positive correlations between nutrient concentrations and pCO_2_ suggested that external inputs affect pCO_2_ in Taihu Lake (Table 1), which is consistent with previous studies carried out in temperate and boreal inland lakes [29,50,51].

Significant negative correlations between S_DO_ and pCO_2_ have also been acknowledged in Taihu Lake (Table 1). S_DO_ reflects the balance between photosynthesis (CO_2_ consumption), respiration (CO_2_ production) activity, and gas exchange at the air–water interface [52]. When S_DO_ > 1, photosynthesis rates are high, and when S_DO_ < 1, respiration rates are high. In this study, S_DO_ was significantly lower in z1 than in z2 and z3 (Figure 2c). In z1, S_DO_ was less than 1 for 65% of samples; when it comes to z2 and z3, S_DO_ was less than 1 for only 10% of samples. It was shown that z1 had higher respiration rates, which caused the CO_2_ level to rise.

Significant negative correlations between Chl–a and pCO_2_ were observed in different zones (Table 1). In eutrophic lakes, algal blooms tend to lower CO_2_ concentrations owing to the high rates of photosynthesis [17,53]. This was consistent with the observed seasonal patterns of Chl–a and pCO_2_ (Figure 4c,d). Higher water temperature stimulated algae growth and bloom formation, which resulted in a decrease in pCO_2_ [53,54]. The higher respiration rates (60% of summertime SDO less than 1), as well as the impact of the inflow rivers, may have contributed to the observation that 80% of summertime CO_2_ was still oversaturated in z1. Summertime in Taihu Lake is commonly characterized by intense cyanobacterial blooms [5,12], which lead to a significant drop in pCO_2_ and the production of enormous amounts of CO_2_. Numerous earlier research has discovered a significant negative correlation between pCO_2_ and Chl-a [53,54,55,56].

Similar temporal variations between annual pCO_2_ and nutrient concentrations in Taihu Lake was observed from 1992 to 2006 (Figure 2 and Figure 5). In addition, significant positive relationships of annual GDP and WD and annual average pCO_2_ were observed from 1992 to 2006 (Figure S3c,f). The large input of nutrients into lakes from WD might promote the mineralization of organic matter and increase CO_2_ concentrations [29]. Anthropogenic organic and inorganic carbon might be deposited into lakes through WD, which promotes CO_2_ concentrations as well [4,57]. Overall, the results of this study highlight the significance of external impacts on CO_2_ concentrations in Taihu Lake.

### 4.3. Response of CO_2_ Dynamics to Eutrophication

There is still a lack of consensus concerning the response of CO_2_ dynamics to eutrophication given contrasting observations among different eutrophic aquatic systems [27,28,53,54,58,59]. Although cyanobacterial blooms are frequent and intensive in Taihu Lake, a hypereutrophic lake, it still acts as a noteworthy CO_2_ source to the atmosphere, which potentially stems from external inputs from its catchment (see above). Considering the contrasting findings regarding the relationship between pCO_2_ and lake eutrophication, the results of this study offer unique insights into how CO_2_ concentrations react to lake eutrophication: lake eutrophication coupled with higher primary productivity can contribute to decreasing CO_2_ concentrations (Table 1), as indicated by the significant negative relationship of pCO_2_ with Chl–a and S_DO_). However, eutrophic lakes can still act as net sinks for CO_2_ due to other processes driven by external inputs. This finding is in line with recent research that has shown how eutrophication can boost or reverse the role of lakes as a source or sink of CO_2_ [23]. Lakes closely interact with their surrounding catchments; changes in land use, hydrology, nutrient inputs, precipitation can affect the CO_2_ concentrations in lakes and may likewise clarify variations in the response of CO_2_ dynamics to eutrophication in different aquatic systems [4,60,61,62]). Consequently, several Chinese government initiatives are initially expected to influence future CO_2_ dynamics in Taihu Lake, including stringent measures to minimize nutrient inputs from point and non-point sources, reconstruction of wetlands surrounding Taihu Lake, and restoration of submerged vegetation. Upcoming studies focusing on CO_2_ dynamics should consider lakes and their catchments if these controversial conclusions are to be reassessed.

### 4.4. Implications and Reflections

The major conclusion of this research is that external pollutant inputs affect not only the nutrient concentrations in Taihu Lake, but also the CO_2_ concentrations. Nonetheless, further research is required to determine the mechanism underlying the high pCO_2_ in the inflow river mouth zone. Specifically, it is unidentified whether CO_2_ is produced directly by inflowing rivers or by the decomposition of organic matter transported by inflowing rivers within lakes. Identifying this mechanism will massively increase our comprehension of the role of lakes in watershed-scale carbon cycling. Future research would benefit from supplementary analysis and discussion of the relationship between CO_2_ dynamics and eutrophication of lakes in the present study, which would help clarify the role of lakes in carbon cycling.

## 5. Conclusions

(1) pCO_2_ and nutrient concentrations were significantly higher in the inflow river mouth zone than in the Meiliang Bay and central lake, indicating that external inputs of nutrients from the catchment substantially affect water parameters in Taihu Lake.

(2) The temporal changes of pCO_2_ and nutrient concentrations (TN, TP, and NH_4_^+^–N) were correlated to the changes in GDP and wastewater discharges of Wuxi City, demonstrating that the water quality of Taihu Lake was sensitive to catchment development activities.

(3) pCO_2_ was negatively correlated with Chl-a and the saturation of dissolved oxygen, but positively correlated with nutrient concentrations (e.g., TN, TP, and NH_4_^+^–N) in Taihu Lake.

Overall, the findings of this study highlight that catchment effects must be considered when modelling and estimating CO_2_ emissions from the heavily human-impacted eutrophic lakes.

## Figures and Tables

**Table 1:** Spearman’s rank correlation coefficient between pCO_2_ and water parameters ^a^.

	TN	TP	NH_4_^+^–N	NO_3_^−^–N	PO_4_^3−^–P	Chl–a	EC	DO	S_DO_
Zone1	0.49 **	0.38 **	0.63 **	−0.24 **	0.48 **	−0.41 **	0.38 **	−0.58 **	−0.64 **
Zone2	0.47 **	0.11 ^ns^	0.53 **	0.33 **	0.30 **	−0.41 **	0.52 **	0.09 ^ns^	−0.33 **
Zone3	0.38 **	0.32 **	0.35 **	0.31 **	0.04 ^ns^	−0.14 ^ns^	0.35 **	0.30 **	−0.05 ^ns^
All data	0.62 **	0.58 **	0.70 **	0.10 ^ns^	0.53 **	−0.16 **	0.50 **	−0.32 **	−0.55 **

^a^ The numbers of each of the water parameters in z1, z2, and z3 are 210, 349, and 170, respectively. The total number of each water parameter is 729. ** Correlation is significant at the *p* < 0.01 level. ^ns^ No significant correlation.

## Data Availability

The data that support the findings of this study will be provided upon reasonable request to the corresponding author. Water physicochemical and biological parameters were acquired from the Lake–Watershed Science Data Centre, National Earth System Science Data Sharing Infra-structure, National Science & Technology Infrastructure of China (http://lake.geodata.cn (accessed on 29 January 2020)).

## Supplementary Materials

The following supporting information can be downloaded at: https://www.mdpi.com/article/10.3390/ijerph191912150/s1, Text S1: Calculations of pCO_2_; Text S2: Calculations for the saturations of CO_2_ and DO; Text S3: Relationships of nutrient concentrations and pCO_2_ with GDP and waste water dis-charge [63,64]. Table S1. Coefficients for the temperature and salinity dependence of solubility of CO_2_; Table S2. The numbers of the parameters included in Figure 2; Table S3. Standardized loadings of each parameter in PCA analysis; Figure S1. The annual average atmospheric pCO_2_ from 1992 to 2006; Figure S2. Gross domestic production (GDP) (a) and wastewater discharge (WD) (b) in Wuxi City (China) from 1992 to 2006; Figure S3. Correlations between GDP and wastewater discharge in Wuxi city and annual mean concentrations of TN (a,d), TP (b,e), and pCO_2_ (c,f), respectively. Data used here are the annual average values of whole dataset during 1992–2006.
