# Supplementary material for: Spatiotemporal Patterns in pCO2 and Nutrient Concentration: Implications for the CO2 Variations in a Eutrophic Lake
Source: Int J Environ Res Public Health. 2022 Sep 25;19(19):12150. doi: 10.3390/ijerph191912150 (PMC9564908; doi:10.3390/ijerph191912150)
Supplement: Supplementary file 1 [file ijerph-19-12150-s001.zip › ijerph-1881183-supplementary.pdf]

Supplementary material

Spatiotemporal Patterns in pCO_2 and Nutrient Concentration: Implications for the CO_2 Variations in a Eutrophic Lake

Jie Xu [1], Zheng Zhou [1], Jie Chen [1], Haihua Zhuo [1], Jie Ma [2,*] and Yunbing Liu [1,*]

1 Yangtze River Basin Ecological Environment Monitoring and Scientific Research Center, Yangtze River Basin Ecological Environment Supervision and Administration Bureau, Ministry of Ecology and Environment, Wuhan 430010, China
2 Nanjing Institute of Environment Sciences, Ministry of Ecology and Environment, Nanjing 210023, China
* Correspondence: majie@nies.org (J.M.); yunbing_liu@163.com (Y.L.)

Text 1. Calculations of pCO_2

The long-term pCO_2 (μatm) in this study were calculated from the water temperature, total alkalinity (TA), and pH. The detailed calculation procedures is as follows:

$$pCO_2 = 1000 \times [(TA + [H^+] - [OH^-])/(a_1 + 2a_2)]/(K_1/[H^+] + K_1 \times K_2/[H^+]^2 + 1)/K_H \quad (S1)$$

where K_1 and K_2 are the first and second dissociation constant in the classical carbonate chemical system, respectively, and were calculated from water temperature (T_w, in K):

$$\log_{10}K_1 = -356.3094 - 0.06091964T_w - 21834.37/T_w + 126.8339 \log_{10}T_w - 1684915/T_w^2 \quad (S2)$$

$$\log_{10}K_2 = -107.8871 - 0.03252849T_w + 5151.79/T_w + 38.92561\log_{10}T_w - 563713.9/T_w \quad (S3)$$

a1 and a2 are the ionization fractions with available concentrations of $[H^+]$ and $[OH^-]$, which were calculated from pH, and are calculated as follows:

$$a_1 = ([H^+]/K_1 + 1 + K_2/[H^+])^{-1} \quad (S4)$$

$$a_2 = ([H^+]^2/(K_1 \times K_2) + [H^+]^2/K_1 + 1)^{-1} \quad (S5)$$

KH is the Herry's constant, and is adjusted by water temperature:

$$\log_{10}K_H = 108.3865 + 0.01985076T_w - 6919.53 / T_w - 40.45154 \log_{10}T_w + 669365 / T_w^2 \quad (S6)$$

Text 2. Calculations for the saturations of CO_2 and DO

For CO_2 saturation:

$$S_{gas} = \frac{C_w}{C_{eq}} \times 100\% \quad (S7)$$

Where S_{gas} is the CO_2 saturation in surface water (%), C_w is the CO_2 concentrations in the water, C_{eq} is the CO_2 concentration in the water that is in equilibrium with their atmospheric concentrations at the surface water temperature, C_w and C_{eq} were calculated according to Wanninkhof. (2014)[1]:

$$\mathrm{Ln}\ \beta = A_1 + A_2(100/T) + A_3\ln(T/100) + S\,[B_1 + B_2(T/100) + B_3(T/100)^2] \quad (S8)$$

Where β is the Bunsen coefficient (μmol L^{-1} atm^{-1}), T is the water temperature in Kelvin (k), S is the salinity in water (‰), A_i *and* B_i are constants listed in Table S1. Taihu Lake is a freshwater lake, here, we supposed S ≈ 0.

Table S1. Coefficients for the temperature and salinity dependence of solubility of CO_2.

Gas	A_1	A_2	A_3	B_1	B_2	B_3
CO_2	−58.0931	90.5069	22.2940	0.027766	−0.025888	0.0050578

The average annual atmospheric pCO_2 were provided by the NOSS/ESRL (http://www.esrl.noaa.gov (accessed on 1 April 2020)), the dynamics of atmospheric pCO_2 from 1992 to 2006 were shown in Fig. S1.

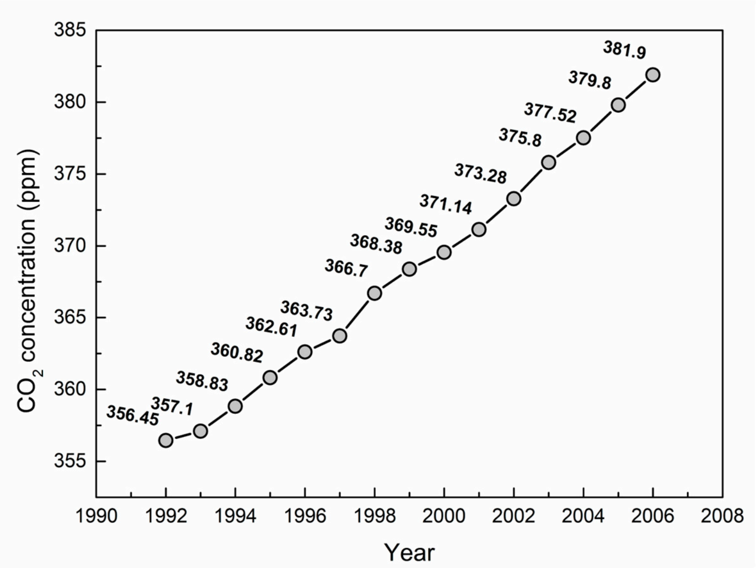

Figure S1. The annual average atmospheric pCO_2 from 1992 to 2006.

For DO saturation calculation:

DO_{sat} in mg L^{-1} was defined according to Benson and Krause Jr. (1984)[2]:

$$DO_{sat} = \exp(-139.34411 + 1.575701\times10^{5}\times T^{-1} - 6.642308\times10^{7}\times T^{-2} + 1.2438\times10^{10}\times T^{-3} - 8.621949\times10^{11}\times T^{-4}) \quad \text{(S9)}$$

$$S_{DO} = \frac{DO_{sat}}{DO_w} \times 100\% \quad \text{(S10)}$$

Where DO_w is the DO concentrations in the water, T is the water temperature in Kelvin (K).

Table S2. The numbers of the parameters included in Fig. 2.

Parameters	pH	TA	SDO	EC	TN	TP	NH_4^+–N	Chl–a
Z1	342	342	321	344	348	347	336	337
Z2	684	691	529	693	695	693	556	672
Z3	342	342	267	343	347	347	281	334

Table S3. Standardized loadings of each parameter in PCA analysis.

Variable	Dim1	Dim2
pH	−0.13	0.13
TN	0.18	−0.03
TP	0.13	0.30
Chl–□	0.01	0.35
DO	−0.10	−0.18
NH_4^+	0.17	−0.01
TA	0.16	−0.11
T	−0.04	0.32
EC	0.13	−0.13
PO_4^{3-}	0.15	0.17
NO_3^-	0.00	−0.29
NO_2^-	0.05	0.00
pCO_2	0.16	0.01

Text 3. Relationships of nutrient concentrations and pCO_2 with GDP and waste water discharge

The GDP and WD in Wuxi City were presented in Fig. S2. Clearly, GDP gradually increased from 1992 to 2001, and rapidly increased between 2001 and 2006. The WD showed a slight decrease from 1998 to 2000, and then rapidly increased from 2000 to 2006.

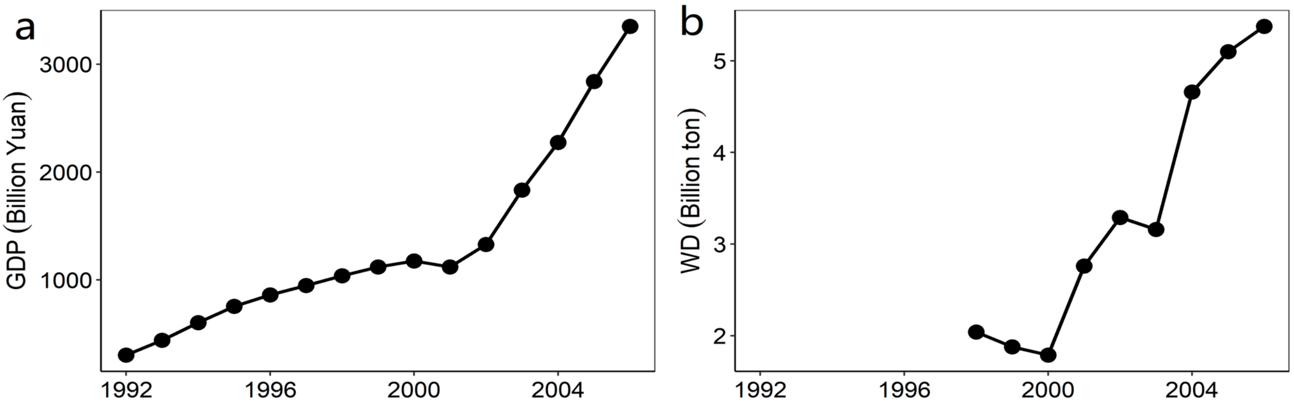

Figure S2. Gross domestic production (GDP)(**a**) and wastewater discharge (WD)(**b**) in Wuxi City (China) from 1992 to 2006.

The correlations between GDP and nutrient concentrations were distinct in 2 periods (1992–1996 and 1996–2006). Annual TN concentration in Taihu Lake was significantly correlated with the annual GDP in Wuxi city (p < 0.01), while TP concentration was only significantly related to GDP during 1992–1996. Moreover, the significant and positive relationship between annual waste water discharges and TN concentration was clear (Fig. S3(**d**), p < 0.01). In addition, the annual GDP and waste water discharges in Wuxi city were significantly and positively correlated with annual pCO_2 in Taihu Lake (Fig. S3(**c**) and (**f**)).

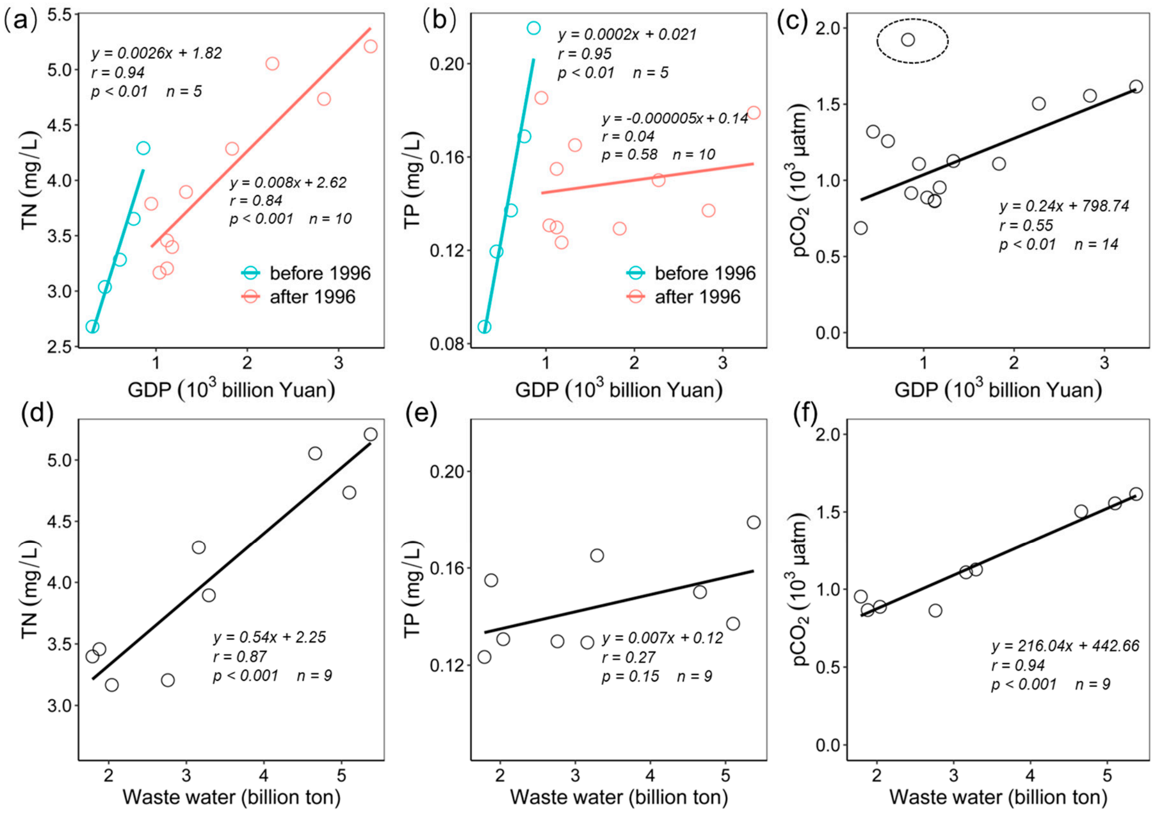

Figure S3. Correlations between GDP and wastewater discharge in Wuxi city and annual mean concentrations of TN (**a,d**), TP (**b,e**), and pCO_2 (**c,f**), respectively. Data used here are the annual average values of whole dataset during 1992–2006.

Reference

1. Wanninkhof, R., Relationship between wind speed and gas exchange over the ocean revisited. *Limnol. Oceanogr. Methods* **2014**, *12*, 351–362. https://doi.org/10.4319/lom.2014.12.351
2. Benson, B.B., Krause, D., The concentration and isotopic fractionation of oxygen dissolved in freshwater and seawater in equilibrium with the atmosphere. *Limnol. Oceanogr*. **1984**, *29*, 620–632. https://doi.org/10.4319/lo.1984.29.3.0620
